# Intrinsically Disordered Proteins: Insights from Poincaré, Waddington, and Lamarck

**DOI:** 10.3390/biom10111490

**Published:** 2020-10-28

**Authors:** Prakash Kulkarni

**Affiliations:** Department of Medical Oncology and Experimental Therapeutics, City of Hope, National Medical Center, Duarte, CA 91010, USA; pkulkarni@coh.org

**Keywords:** intrinsically disordered proteins, conformational dynamics, noise, phenotypic switching, MRK hypothesis, evolutionary transition, multicellularity

## Abstract

The past quarter-century may justly be referred to as a period analogous to the “Cambrian explosion” in the history of proteins. This period is marked by the appearance of the intrinsically disordered proteins (IDPs) on the scene since their discovery in the mid-1990s. Here, I first reflect on how we accidentally stumbled on these fascinating molecules. Next, I describe our research on the IDPs over the past decade and identify six areas as important for future research in this field. In addition, I draw on discoveries others in the field have made to present a more comprehensive essay. More specifically, I discuss the role of IDPs in two fundamental aspects of life: in phenotypic switching, and in multicellularity that marks one of the major evolutionary transitions. I highlight how serendipity, imagination, and an interdisciplinary approach embodying empirical evidence and theoretical insights from the works of Poincaré, Waddington, and Lamarck, shaped our thinking, and how this led us to propose the MRK hypothesis, a conceptual framework addressing phenotypic switching, the emergence of new traits, and adaptive evolution via nongenetic and IDP conformation-based mechanisms. Finally, I present a perspective on the evolutionary link between phenotypic switching and the origin of multicellularity.

## 1. Introduction

“A calm and humble life will bring more happiness than the pursuit of success and the constant restlessness that comes with it.”

-Albert Einstein

I begin by wishing *Biomolecules* a Happy 10th Anniversary and by thanking the Editorial Management Team for the invitation to contribute with this reflective essay. In 2018, I was invited by Prof. Vladimir (Volodya) Uversky to join the Editorial Team of *Biomolecules* as Associate Editor-in-Chief. It was a big honor for me given the Journal’s pre-eminence and its rising status amongst competitors. At the same time, I also realized it was a huge responsibility. It has been a pleasure to work with Volodya and all the members of the Editorial Team in Basel. Over the past three years, we have coedited special issues, contributed articles together, and issued the daunting Janus Challenge [1]. Perhaps, in the not too distant future, we will have the pleasure and honor to greet the intellectual(s) who will meet this challenge and have their work published in *Biomolecules*. Going forward, we envision *Biomolecules* to publish more interdisciplinary and cutting-edge papers, especially in quantitative biology across multiple spatiotemporal scales, to ensure that the Journal not only remains competitive, but also remains ahead of the pack. Here, I reflect on my career in science that began almost 45 years ago and covers many disciplines and scales.

Unlike many of my brilliant colleagues who majored in physics and mathematics, my undergraduate degree was in zoology with a minor in chemistry. Studying the origin and evolution of life on earth and learning about the appearance of the bilateria and the ensuing Cambrian “explosion” was truly exciting. Among the chemistry classes, although I found the lectures on physiological chemistry quite interesting, I was awed by a professor who taught us physical chemistry; in particular, I was fascinated by his lectures on thermodynamics. He was especially interested in protein chemistry and the physical basis of protein folding. Although at the time (in the early 1970s) it was common knowledge that structure defines protein function [2], how a protein molecule adopts a particular conformation from virtually endless possibilities in a relatively short, biologically relevant time scale (the Levinthal paradox) was perplexing [3]. I was thus infatuated by proteins. However, I never imagined back then that I would end up working on elucidating pattern formation during early embryonic development of the fruit fly, a model for bilateria, or on proteins that *appear* to lack structure*!

After completing my master’s degree also in zoology, I decided to pursue graduate studies in biochemistry. For my PhD thesis, my mentor, S.N. Hegde, suggested that I purify and characterize two disaccharidases; namely, sucrase.isomaltase and maltase.glucoamylase from pigeon intestinal epithelial cells (enterocytes). The two enzyme complexes purified as high-molecular-weight protein complexes of ~200 kDa and analogous to their human orthologues, upon denaturation, disassociated into two polypeptides representing the individual enzyme activities [4]. Subcellular fractionation studies indicated that they localized to the “brush border” (the apical surface) of the enterocytes [5]. However, it was unclear whether they are synthesized as a single polypeptide from a gigantic transcript or are synthesized by two independent mRNAs and then assembled into a complex on the apical surface. 

Blobel and Dobberstein had just published their seminal work [6,7] providing compelling evidence for the “signal hypothesis” that Blobel and Sabatini had advanced earlier [8]. The fact that they did this by reconstituting the translation/translocation system in vitro was, to me, truly amazing and, quite frankly, very appealing. Thus, I was very excited to discern how the two disaccharidase complexes were synthesized and delivered to their final destination using their approach.

At the time I was a visiting scholar in J. Ganguly’s laboratory in the Department of Biochemistry at the Indian Institute of Science (IISc) and was assigned to work with M.R.S. Rao. Rao was well-versed with the wheat germ and rabbit reticulocyte in vitro translation systems. I expressed my interest to Rao and he readily agreed to help me. Unfortunately, just as I was getting ready to set up the cell-free protein synthesis assay, Semenza and colleagues published a brief report demonstrating that at least sucrase.isomaltase was indeed synthesized as a single polypeptide and inserted into the brush border membrane via an N-terminal hydrophobic sequence [9]. I was highly disappointed.

Nonetheless, the structure/function paradigm and protein folding continued to awe me, and I pondered pursuing this fascination as I thought about postdoctoral studies. However, I was concerned that my lack of a solid background in physics and mathematics might preclude me from pursuing a career in biophysical chemistry in the future. I even considered, albeit for a fleeting moment, applying for a postdoctoral position with Buzz Baldwin at Stanford but decided not to. Instead, taking the microsomal translocation machinery apart and reconstituting it in vitro to elucidate the molecular mechanism, by which a nascent polypeptide is inserted into the endoplasmic reticulum and is ultimately delivered to its final destination, seemed more tractable with the training I had obtained in biochemistry. The lure of discovery was strong, and I decided that I would pursue my postdoctoral studies in a laboratory that was among the leaders in the field. At the time, the choices were obvious: Günter Blobel’s laboratory at the Rockefeller, Ben Dobberstein’s group at the EMBL in Heidelberg, or David Sabatini’s laboratory at NYU. 

I was fortunate to be accepted by Sabatini. I arrived in New York in January 1985 and was immediately assigned to work in Gert Kreibich’s group. Thus, I began my postdoctoral training with the cDNA cloning and sequencing of the ribophorins together with Victoria Harnik-Ort, a graduate student, and Dvorah Ish-Shalom, a postdoctoral fellow. The Sabatini laboratory was the epitome of cell biology. It was a truly international laboratory filled to the rafters with many bright young people and visiting faculty from all over the world. There I spent four years and had the opportunity to meet and hear many famous scientists when they visited Sabatini including Günter Blobel, Ben Dobbertsein, Christian de Duve, George Palade, and Cyrus Levinthal, among many others. Subsequently, I ran into Blobel a few times. However, the awe-inspiring tour of his laboratory he gave me, and watching him as he talked passionately about the signal hypothesis while we were seated in his office, left an indelible impression; even to this day, I have a very vivid recollection of that particular visit and his parting advice, “Remember, beautiful ideas sometimes can die from ugly facts; but, don’t be afraid to follow your gut,” still rings in my ear. Rockefeller University is one of my most revered institutions. Hence, the opportunity to not only visit this Mecca of cell biology that was home to giants like George Palade, Christian de Duve, Albert Claude, Phil Siekevitz, Keith Porter, David Sabatini, and many others, but to also have the good fortune to meet with Gunter Blobel, was a singularly humbling experience for me.

Although I had a reasonably productive period and learned a lot in the Sabatini/Kreibich laboratory, I did not get the feeling of meeting my tryst. However, as destiny would have it, one evening, my wife and I went to the Rockefeller University to attend a Harvey Lecture by Walter Gehring on the *Drosophila Antennapedia* homeotic gene held in the iconic Caspary auditorium and were awed beyond our wildest imagination. Our excitement was palpable to the folks around us on the bus we rode home that evening and it culminated in our decision to work on developmental biology. So, in early 1989, my wife and I left New York and arrived in Pasadena to join the Eric Davidson and Carl Parker groups, respectively, at Caltech. In the Parker laboratory, I had the opportunity to meet Walter Gehring when he visited Carl and show him our data on the expression patterns of the *Drosophila Oct* genes during early embryonic development that transcended the boundaries of genetically defined segmentation genes on the fly [10]. Interestingly, several decades later, in 2010, I also had the opportunity to meet Buzz Baldwin at the first Gordon Research Conference on intrinsically disordered proteins (IDPs) held in Davidson College, North Carolina. 

## 2. Intrinsically Disordered Proteins and Phenotypic Switching

After more than 20 years that included my sojourn through NYU, Caltech, and Yale, and the biotech industry, I finally had the opportunity to start my own laboratory. Robert Getzenberg had just joined the Brady Institute of Urology at Johns Hopkins University as the Donald Coffey Professor and Research Director. Rob called me one day and asked me if I would be interested in joining the Brady and continuing our collaboration on prostate cancer (PCa)/BPH that we had begun when I was in the industry. An interesting finding that had ensued from that collaboration was the identification of Prostate-associated Gene 4 (PAGE4; back then referred to as JM-27), although we had no clue regarding its role in the diseased prostate where it was upregulated [11]. Yu Zeng, a very bright postdoc (now at China Medical University), did a lot of the initial work on the PAGE4 biology at Hopkins.

At Hopkins, my research focused on the functions of this rather intriguing protein and two serendipitous discoveries provided the breakthroughs and critical insights that fostered our scientific research. The first discovery was that PAGE4 is a highly intrinsically disordered protein (IDP) [12]. I had implored Pamela Bjorkman at Caltech to help with the crystallization. However, after more than six months of relentless efforts by her graduate student Fan Yang, Pam informed me that PAGE4 is not amenable to crystallization. I felt hopeless—my dreams of securing funding to support our research seemed shattered. I reached out to a few structural biologists to find out what can be done if a protein is non-crystallizable and was quite intrigued by an email from Gaetano (Guy) Montelione (Rutgers) that read, “Perhaps your protein may be *intrinsically disordered.*” 

This opened our eyes to a whole new world about which we knew nothing and forced us to think hard in ways that we would have never anticipated or envisioned. This was further fueled by reports that ~80% of cancer-associated proteins are IDPs including the most commonly encountered oncogenes [13,14] and bioinformatics analyses suggesting that >90% of the Cancer/Testis Antigens [15], and the “Yamanaka factors” that actuate the reprogramming of somatic cells to embryonic stem cells [16], are predicted to be IDPs [17]. Together, these observations raised the possibility in my mind that IDPs are somehow involved in state (phenotypic) switching.

## 3. The MRK Hypothesis 

I was very fortunate that, at the time, several brilliant students happened to work with me and contributed so much to our understanding of the IDPs and their role in phenotypic switching. In particular, Gita Mahmoudabadi (now at Stanford), who had just completed her bachelor’s degree from Georgia Tech and was en route to Caltech as a fresh graduate student, decided to spend a summer in my laboratory as a SURE Fellow, and Krithika Rajagopalan (now at UCSD), who worked with me while she was a Master’s student at Johns Hopkins, and later went to Columbia University as a graduate student, deserve a lot of credit. The numerous exciting, and at times enthralling, discussions we had in the Brady, and the several insightful exchanges we had with Govindan Rangarajan, a mathematician in IISc, ultimately led us to propose how IDPs could cause phenotypic switching in a nongenetic manner [18].

Back then, it was evident that IDPs play important roles in many biological processes, and in many cases, the transition from disorder to order upon binding to their targets (also referred to as coupled folding and binding) [19,20,21]. Moreover, it was generally held that while in some cases an ordered conformation is induced by the interacting partner, a phenomenon referred to as “induced fit,” in other instances, the IDP ensemble samples multiple conformations a priori and the ligand selects the most favored prefolded state from these conformations [22]. Furthermore, it was observed that some IDPs can shift the overall conformation of their ensembles while remaining disordered [23]. Lastly, it was demonstrated that, with multiple conformational states and rapid conformational dynamics, IDPs engage in a myriad of, and often “promiscuous,” interactions [24,25]. Interestingly, it was also suspected that there is “noise” in signaling networks that contributes to cellular decision making. However, the origin of this noise and how it shapes cellular outcomes had remained poorly understood, although it was suggested that this noise results from the intrinsic promiscuity of protein–protein interactions [26].

**Figure 1:**
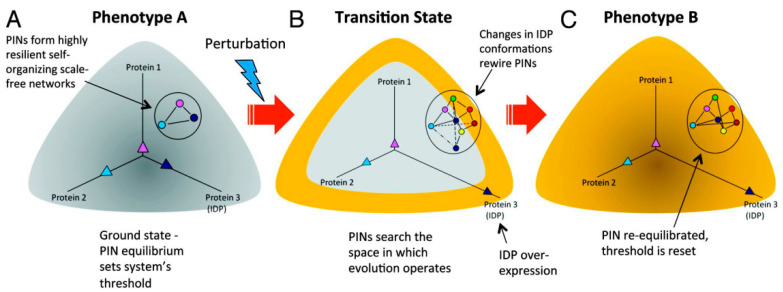
Rewiring of protein networks facilitates state-switching by activating latent pathways. (**A**) The state of a cell with phenotype A is depicted in grey and shows a simple protein network with three proteins (1‒3), of which one is an IDP (indicated in dark blue) and expressed at different levels represented by the three vectors. This configuration represents the protein network’s ground state threshold. (**B**) Depicts a transition state. A perturbation causes increased IDP expression (protein 3). Overexpression of the IDP results in promiscuity and the protein network explores the network search space shown by the various dashed lines. This transition state is depicted state in yellow around the grey area. (**C**) The state of the cell after it has transitioned to phenotype B from phenotype A represented in yellow. A particular configuration of the protein network that increased its fitness is “selected,” which now represents the new ground state. Adopted from [18].

Therefore, we postulated that, since IDPs can populate multiple conformational states and exhibit rapid conformational dynamics, stochastic interactions between IDPs and their partners can give rise to noise and referred to it as “conformational noise” to emphasize that it is distinct from transcriptional noise [27,28]. Furthermore, we enunciated that the collective effect of conformational noise is an ensemble of the configurations of cellular protein interaction networks (PINs) from which the most suitable can be explored in response to perturbations, conferring protein networks with remarkable flexibility and resilience. The ubiquitous presence of IDPs as transcriptional factors, and more generally as hubs in protein networks, further supported their role in the propagation of transcriptional (genetic) noise. We implied that, as effectors of transcriptional and conformational noise, IDPs can rewire protein networks and unmask latent interactions in response to perturbations. Thus, we posited that noise-driven activation of latent pathways can drive state-switching events such as cellular transformation in cancer (Figure 1). 

**Figure 2:**
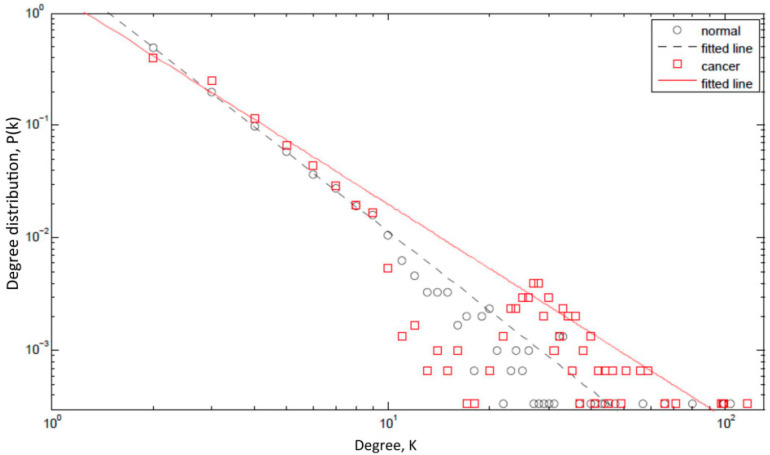
Degree distribution plot. The figure shows the probability P(k) that a given protein interacts with k other proteins (the so-called degree distribution) on a log-log scale. The figure compares the degree distribution of a normal protein regulatory network (black circles) with that of a network impacted by cancer (red rectangles). A majority of the hubs in protein networks are IDPs and these IDPs have aberrant expression profiles in cancer and, moreover, they preferentially interact with other hubs. Consequently, the slope of the straight line fitted to the points for a cancer network (red solid line) is smaller than that for a normal network (black dashed line). Further, the maximum degree increases in a cancer network (the red rectangles extend further to the right as compared to the black circles). All simulations were carried out using Matlab (MATLAB version 7.12.: The MathWorks Inc., 2011). Adopted from [1].

To test this hypothesis, we created a model of a protein network with the topological characteristics of a cancer protein network and determined its response to perturbation in the presence of IDP hubs and conformational noise (Figure 2). We observed several interesting results, such as an increase in the average degree per node, increase in the maximum degree, and a decrease in the power law decay factor γ. However, the most interesting observation was the increased resilience of the network to random perturbations [18]. Because numerous IDPs are found to be epigenetic modifiers and chromatin remodelers, we hypothesized that they could further channel noise into stable, heritable genotypic changes. Although in the original enunciation we focused on cancer, it had not escaped our notice that the hypothesis had much broader implications. Therefore, we wrote that, “*Although we have focused on cancer, our thesis is not restricted to cancer and may be more generally applicable to address state-switching in biology*” [18]. 

Thus, it followed that cellular transformation and the attendant properties of cancer cells that ensue, which are also driven by IDP dysregulation, represent an example of adaptive evolution embodied in Lamarck’s ideas of inheritance of acquired characteristics [29]. However, I must emphasize that, as appealing and as elegant as it seemed, evidence supporting the critical aspects of the hypothesis was lacking or weak or indirect at best. Although I was reluctant at first because I felt it may sound too egoistic, we finally followed suit of a Wikipedia article on state switching (https://en.wikipedia.org/wiki/State_switching) and referred to it as the MRK hypothesis after the preceptors Mahmoudabadi, Rangarajan, and Kulkarni [30]. In retrospect, it is gratifying to see how the MRK hypothesis that provided a simple yet elegant framework for phenotypic switching in general, and a nongenetic mechanism in cancer in particular, has facilitated further progress, and as we shall see below, although many details have been elucidated, its fundamental aspects are still in place. However, the unequivocal demonstration of conformational noise (which is currently lacking) could be one of the most significant discoveries in this field—the first area of future research among the six areas I identify in this essay.

## 4. PAGE4 Is a Transcriptional Regulator

The second serendipitous discovery was that PAGE4 potentiates c-Jun transactivation and was made by Krithika [31]. To identify PAGE4 interacting partners, we employed the yeast two-hybrid system in collaboration with Ajay Bhargava (Shakti Biosciences). One of the proteins that we identified in this screen was ZNF394, a 64 kDa zinc finger protein. Overexpression of ZNF394 in COS-7 cells was shown to inhibit the transcriptional activities of c-Jun and AP-1 reporters, suggesting that ZNF394 is a transcriptional repressor in the MAP kinase signaling pathway [32]. To confirm the yeast two-hybrid data, Krithika set up the c-Jun/AP-1 reporter system in the PC3 PCa cell line. She found that GAL4-c-Jun, in the presence of MEKK, showed robust activation of the reporter gene. However, in contrast to the observed effects in COS-7 cells [32], ZNF394 did not repress c-Jun activity in PC3 cells. Surprisingly, however, the addition of PAGE4 further enhanced the activity of c-Jun, suggesting that PAGE4 may directly interact with c-Jun instead! Finally, we had found a function of PAGE4 that was consistent with its putative DNA-binding ability and the presence of a nuclear localization signal in the N-terminus. We were ecstatic! However, while these observations implied that PAGE4 may interact with c-Jun, we could not rule out the possibility that the interaction may be indirect involving yet another protein/transcriptional regulator. Secondly, we were curious to know if the PAGE4 molecule transitioned from disorder to order upon interacting with c-Jun if indeed it directly interacted with it. 

## 5. Probing the PAGE4 Ensemble Applying Biophysics

To this end, we resorted to single-molecule Förster Resonance Energy Transfer (sm-FRET) microscopy. A unique advantage of this sophisticated technique is that it can capture information normally lost through ensemble averaging of heterogeneous and dynamic samples. Furthermore, the immobilization of single molecules such that they retain their biological activity allows for extended observation of the same molecule, facilitating the capture of slow conformational transitions or binding/unbinding cycles [33,34,35]. Finally, the use of an open geometry for immobilization facilitates direct observation of the response to changing solution conditions or adding ligands. Thus, we began a collaboration with Keith Weninger (North Carolina State University), a brilliant biophysicist. Ruoyi Qiu (now at Stanford University), a highly talented graduate student in Keith’s laboratory, and Krithika engineered cysteine residues near the N-terminus or C-terminus of the 102 amino acid long PAGE4 molecule at positions 18 or 102 by replacing an alanine (A18C) and proline (P102C) residue, respectively. These mutants, alternately combined with the single native C residue at position 63 to generate two double C constructs, were simultaneously labeled with Alexa Fluor 555 (FRET donor) and Alexa Fluor 647 (FRET acceptor) resulting in the random attachment of the donor and acceptor on the cysteines. Protein molecules emitting fluorescence indicating that exactly one donor and one acceptor were isolated for further analysis. Briefly, using labeled PAGE4 that was liposome-encapsulated or surface-tethered, we were able to demonstrate that (i) PAGE4 is highly disordered (ii) in contrast to some other IDPs, no slow conformational switching was observed in the PAGE4 ensemble, (iii) PAGE4 directly interacts with c-Jun and undergoes conformational changes, and (iv) the interaction of PAGE4 with c-Jun is mediated via the proximal portion of the molecule that harbors the transactivation domain [31]. 

Since IDPs are relatively subject to greater post-translational modifications, particularly phosphorylation, than ordered proteins [36,37], Steve Mooney, an extremely bright and imaginative postdoc, decided to interrogate the phosphorylation status of PAGE4. Not surprisingly, he was not only able to demonstrate that, indeed, PAGE4 is phosphorylated in PCa cells, but was also able to identify the specific S/T residues as well as the kinases that are responsible for the phosphorylation. More specifically, he was able to demonstrate that the stress-response kinase, Homeodomain-Interacting Protein Kinase 1 (HIPK1), phosphorylated PAGE4 predominantly at T51 and that this was critical for c-Jun transactivation [38]. A mutant form of PAGE4, in which T51 was replaced with an alanine residue (T51A) that was not phosphorylated by HIPK1, failed to transactivate c-Jun. Subsequently, Steve identified a second kinase, CDC-like Kinase 2 (CLK2), that hyperphosphorylated PAGE4, and in collaboration with Ajay, we showed that the hyperphosphorylation of PAGE4 attenuated c-Jun transactivation. Interestingly enough, working with Luciane Kagohara (now at Johns Hopkins), who was a postdoc in Robert Veltri’s laboratory in the Brady, we found that while HIPK1 is expressed in both androgen-dependent and androgen-independent PCa cells, CLK2 and PAGE4 are expressed only in androgen-dependent cells [39]. 

Although smFRET is a powerful technique, it has limited resolution power. Therefore, to gain additional insight at the single amino acid resolution, we collaborated with John Orban (University of Maryland), an outstanding expert in protein NMR. Consistent with the cellular data, biophysical measurements employing smFRET, NMR, and small-angle X-ray scattering (SAXS) measurements done by Alex Grishaev (University of Maryland) revealed that HIPK1-PAGE4 exhibits a relatively compact conformational ensemble that binds AP-1, whereas CLK2-PAGE4 is more expanded and resembles a random coil with a diminished affinity for AP-1. Furthermore, in paramagnetic relaxation enhancement (PRE) studies using nonphosphorylated and HIPK1-PAGE4 spin-labeled with (1-oxyl-2,2,5,5-tetramethylpyrroline-3-methyl)methane-thio-sulfonate (MTSL), John’s group probed the molecule for long-range intramolecular interactions. Conservative PRE and nuclear Overhauser effect (NOE) restraints were then used as inputs for calculating ensemble conformations for both WT PAGE4 and phosphorylated PAGE4. Although all restraints are unlikely to be satisfied simultaneously in a single polypeptide chain, the resulting models provided a useful framework for visualizing preferred states of the highly flexible ensemble [39,40]. 

**Figure 3:**
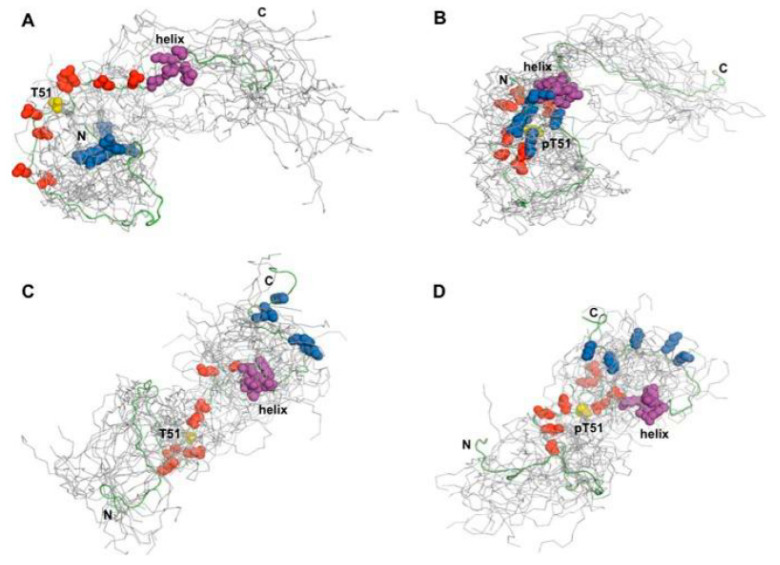
Model for phosphorylation-induced conformational ensemble switching in PAGE4. (**A**), the non-phosphorylated PAGE4 adopts preferred transient structures such as the one highlighted from an ensemble of the 20 lowest energy conformers, where, on average, the N-terminal basic motif (*blue spheres*; Arg-4, Arg-6, Arg-8, Arg-10, and Arg-12) interacts weakly with the central acidic region (*red spheres*; Glu-43, Glu-47, Glu-49, Glu-55, Glu-56, Glu-60, and Asp-62) neighboring Thr-51 (*yellow*). (**B**), upon phosphorylation at Thr-51, the central region becomes more compact and more negatively charged, decreasing the average distance between Thr(P)-51, the basic motif, and the transient helix (*magenta*). (**C**) and (**D**), models of the transient interaction between the central acidic region and the C-terminal basic motif (*blue spheres*; Lys-82, Lys-84, Lys-90, Lys-93, and Lys-95) in non-phosphorylated PAGE4 (**C**) and Thr(P)-51 PAGE4 (**D**). The total number of distance restraints used was as follows: (**A**), 51; (**B**), 55; (**C**), 53; (**D**), 61. Adopted from [40].

We calculated long-range interactions between the central acidic region and the N- and C-terminal contact sites separately and found that WT PAGE4, on average, populates conformations where the highly basic N-terminal motif (residues 4-12) is within 25 Å of the central acidic region (residues 43-62) neighboring the C63 residue used for attaching the MTSL moiety. These data also showed that phosphorylation at T51 increases the negative charge in this acidic region and induces turn-like structures that provide a more compact transient interaction with the N-terminal motif. In addition, other interactions between a C-terminal motif centered on residue Asn-88 and the transient helix contribute to the overall conformational ensemble. We inferred that these long-range contacts may also be at least partly due to electrostatic interactions of several basic residues in this region (residues 82-95) with central acidic amino acids. Notably, both the N- and C-terminal contacts to the central acidic region decreased accessibility to the transient helix [40]. Taken together, the results suggested that the phosphorylation-induced conformational dynamics of PAGE4 may play a role in modulating changes between PCa cell phenotypes (Figure 3). 

## 6. Molecular Dynamics Simulations (MDS) Corroborated the Dynamic Intramolecular Interactions of the PAGE4 Ensemble

**Figure 4:**
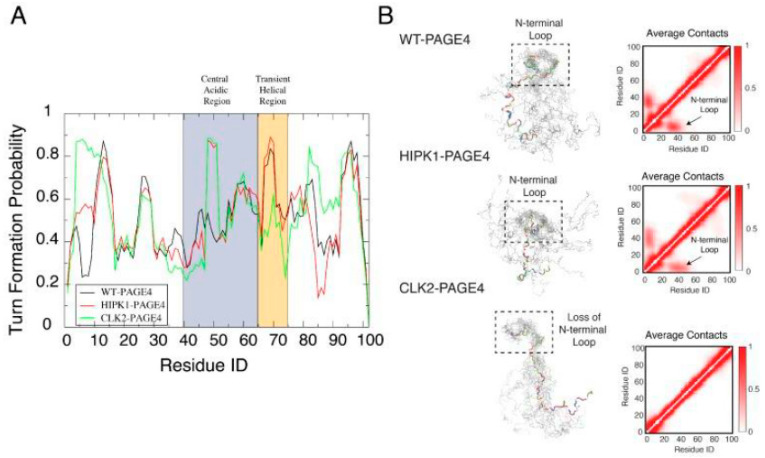
Orderly features behind the disordered PAGE4 ensembles. (**A**) The probability for each residue of PAGE4 to adopt a turn-like structure upon different levels of phosphorylation. The central acidic region and transient helical region are shaded in blue and orange, respectively. The secondary structure was calculated using the Stride algorithm based on the simulated trajectories [45]. Phosphorylations stabilize the turn-like structure in the central acidic region of PAGE4, while hyper-phosphorylation decreases the degree of order in the transiently helical region. (**B**) (**Left**) Representative structural snapshots collected from our simulations generated by AAWSEM. Randomly picked structures are aligned to minimize the root-mean-square deviations (RMSDs) among their N-motifs [46]. (**B**) (**Right**) The average contact maps generated from the simulated ensembles. Contacts are defined as two residues in close spatial proximity to each other. The color bar shows the probability of contact formation. There are non-zero probabilities of contacts formed between the N-motif and the central acidic region in WT-PAGE4 and HIPK1-PAGE4 (indicated by arrows in plots), indicating a metastable structural loop formation in this region. Hyper-phosphorylation eradicates this loop formation in the CLK2 form. Adopted from [41].

More in-depth computational studies to elucidate the interactions underlying the conformational transitions were done together with Xingcheng Lin, a brilliant graduate student in Jose Onuchic’s laboratory at Rice University (now at MIT), and Susmita Roy, a talented postdoc also in Jose’s group (now at IISER, Kolkata) [41,42]. The MD simulations were done using the Atomistic, Associative memory, Water mediated, Structure and Energy Model (AWSEM), a multiscale molecular model that combines atomistic and coarse-grained simulation approaches to elucidate the conformational dynamics of PAGE4 and how its motions change in its different phosphorylated ensembles. The simulations quantitatively reproduced our experimental observations and revealed how structural and dynamical ordering are encoded in the sequence of PAGE4 and how they can be modulated by differential phosphorylation by HIPK1 and CLK2. Furthermore, the simulations uncovered a hidden layer of order underlying the apparent disordered features of PAGE4. They indicated a change in the preference for forming turn-like structures in the central acidic region of PAGE4 upon different levels of phosphorylation (Figure 4). This structural change was consistent with the observations from the NMR experiments we described previously [40]. 

**Figure 5:**
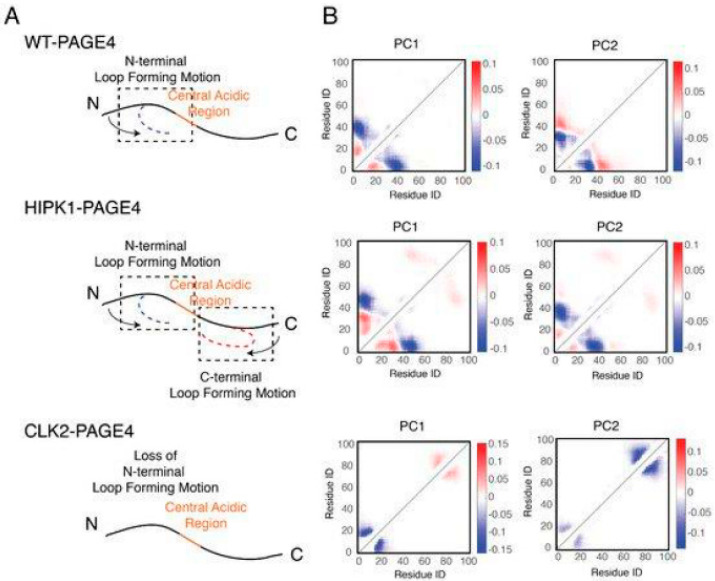
The collective motions revealed from the principal component analysis of PAGE4 simulations are shown. (**A**) Representative cartoon summarizes the collective motions of different phospho-forms of PAGE4. (**Top**) WT-PAGE4 has a collective motion of contacts formed between the N-terminal end and the central acidic region, resulting in a regulated loop formation. (**Middle**) In addition to that, HIPK1-PAGE4 has another loop motion in the C-terminal end that is anti-correlated with that in the N-terminus. (**Bottom**) Hyper-phosphorylation causes the loss of N-terminal loop motion in CLK2-PAGE4. (**B**) The top two principal component modes generated by the contact-based principal component analysis. We plot the coefficients of the first two principal components PC1 and PC2. Larger coefficients indicate a more significant variation of contact formation in that specific principal mode. The relative sign (shown in colors) of two coefficients corresponds to either correlated (same sign) or anti-correlated (opposite signs) formation of contacts. Here, in HIPK1-PAGE4, the C-terminal loop formation has an anti-correlated behavior compared with the N-terminal loop formation. When PAGE4 becomes hyper-phosphorylated, CLK2-PAGE4 loses both N- and C-terminal motion in the first two principal modes. Adopted from [41].

In addition to the residual structural order, the organized dynamics of PAGE4 were also discerned by a principal component analysis (PCA) of the AAWSEM simulations. The analysis (Figure 5) shows that there are correlated motions at the N-terminal half of WT-PAGE4, where the positively charged N-motif forms a loop with the central acidic region of the protein (shown as blue blobs of the first two principal modes in the top panel of Figure 5B). These results revealed that when WT-PAGE4 becomes phosphorylated by HIPK1, the molecule acquires a second type of motion involving loop formation in the C-terminus (C-motif, residues 82 to 95). The C-terminal motion is anticorrelated with the movement of the N-terminus (shown as additional red blobs of the first two principal modes in the middle panel of Figure 5B). This anticorrelation suggested that the two termini take turns forming a loop with the central acidic region of HIPK1-PAGE4. However, upon hyperphosphorylation by CLK2 (CLK2-PAGE4), overall disorder increases accompanied by a loss of both types of correlated motions, except for the correlated local motions among these residues that are close in sequence. This was reflected by randomization of the long-range PC pattern (bottom panel of Figure 5B). Based on these observations, we inferred that the motions associated with the formation of loops by the N- and C-termini of the WT- and HIPK1-PAGE4 may facilitate the binding of PAGE4 to its cognate DNAs or the AP-1 protein complex (or both). Thus, the structural plasticity of N- and C-termini enlarges the scope of interactions for PAGE4 to find its binding partners, while the looping motion assists in the ensuing binding processes. Such a mechanism is reminiscent of the “fly-casting” motion frequently observed in IDPs where plasticity allows them to enlarge their scope of interactions and lowers the free energy barriers for IDPs for finding their binding partners [43,44]. However, consistent with our NMR, and SAXS results [39], in collaboration with Ajay we observed that, upon hyperphosphorylation, CLK2-PAGE4 loses its ability to approach and bind to its transactivation partners, resulting in the loss of function and rapid degradation of PAGE4 [39].

## 7. Oscillatory Dynamics of the PAGE4-Regulated Circuit Drives Phenotypic Switching in PCa Cells 

Much of the work on CLK2 and the ensuing conformational dynamics of the different PAGE4 phospho-forms was undertaken following my move to the University of Maryland. From the frequent and stimulating discussions John and I had, it became obvious that while the biochemical and biophysical studies afforded incredible insight into the conformational dynamics of the various PAGE4 ensembles, and we were able to connect the dots and conjecture how the PAGE/AP-1 interaction may regulate the switch of a PCa cell from an androgen-dependent to an androgen-independent phenotype, we needed a model that could be falsified. 

**Figure 6:**
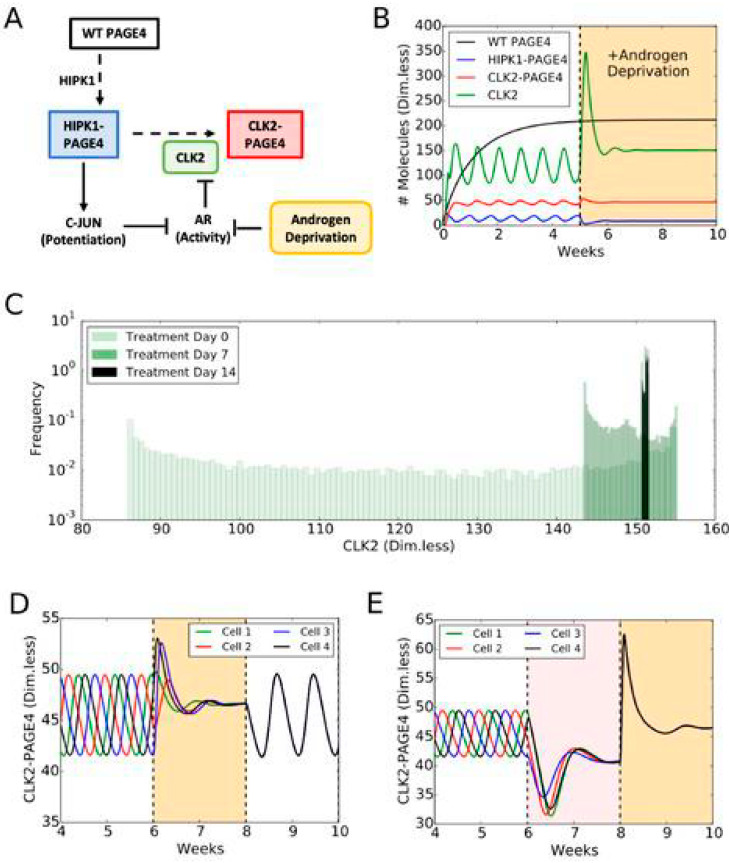
PAGE4 conformational switching gives rise to cell phenotypic oscillations which are suppressed by Androgen Deprivation treatments. (**A**) The PAGE4 phosphorylation circuit and its connection with androgen receptor (AR) activity. Wild-type PAGE4 is double-phosphorylated at two residues by HIPK1 kinase, and HIPK1-PAGE4 is hyper-phosphorylated by the CLK2 kinase. CLK2 is downregulated by AR, which in turn is inhibited by HIPK1-PAGE4 via the intermediates c-Jun. Androgen Deprivation treatment is introduced as an inhibitory signal on AR activity. (**B**) Temporal dynamics of the cellular level of WT PAGE4, HIPK1-PAGE4, CLK2-PAGE4 and CLK2. Without androgen-deprivation therapy (ADT), the oscillatory behavior exhibits a period of approximately one week (left area without shading). ADT (orange-shaded area) quenches oscillations within approximately two weeks. WT PAGE4, HIPK1-PAGE4, CLK2-PAGE4 and CLK2 are represented in dimensionless units. (**C**) Distribution of CLK2 intracellular levels in a simulated cohort of 10,000 prostate cancer (PCa) cells. In the absence of treatment, the distribution of CLK2 levels is broad (“Day 0” case). One week of treatment considerably shrinks the distribution (“Day 7” case). After two weeks of treatment, all cells have a similar level of CLK2 (“Day 14” case). (**D**) Temporal dynamics of CLK2-PAGE4 in four initially unsynchronized cells under intermittent ADT. The orange shading represents the periods of ADT. (**E**) Temporal dynamics of CLK2-PAGE4 in four initially unsynchronized cells under the BAT. The pink and orange shadings represent the periods of AR overexpression and ADT, respectively. Adopted from [41].

Thus, in collaboration with Herbie Levine, a brilliant physicist, and Mohit Kumar Jolly (now at IISc), a phenomenal graduate student in Herbie’s group at Rice University, we modeled the circuit employing the tools of nonlinear dynamics. The model considered the three physiologically relevant PAGE4 conformational ensembles, namely, WT-PAGE4, HIPK1-PAGE4, and CLK2-PAGE4, and the enzymes catalyzing the reactions that convert WT-PAGE4 into the HIPK1-phosphorylated and CLK2-phosphorylated forms. Furthermore, since c-Jun potentiation can indirectly increase the level of CLK2, a negative feedback loop is included in the model. Thus, the model showed that the levels of HIPK1-PAGE4 and CLK2-PAGE4 can exhibit oscillatory behavior and, hence, AR activity. This simple yet elegant model demonstrated how differential phosphorylation of PAGE4 can lead to transitions between androgen-dependent and androgen-independent phenotypes by altering the AP-1/androgen receptor regulatory circuit in PCa cells. Thus, we postulated that the intracellular oscillatory dynamics of HIPK1-PAGE4, CLK2-PAGE4, and AR activity results in phenotypic heterogeneity in an isogenic cell population [39,41,42] (Figure 6). Consistent with this model, single cells isolated from a population of androgen-dependent LNCaP PCa cells exhibited varying degrees of androgen dependence when grown in an androgen-depleted medium [47]. Our work on another IDP, c-MYC, an oncoprotein that was found to reversibly cause hepatocytes to switch from a malignant phenotype to nonmalignant one by simply dialing up or dialing down its expression levels [48], in collaboration with Nivedita Rangarajan, a very bright undergraduate student (now at Princeton), and Abhyudai Singh (University of Delaware), is yet another example of how an IDP may nongenetically regulate phenotypic switching [49].

## 8. Phenotypic Plasticity and Cell Fate Decisions in Cancer: Insights from Waddington’s Epigenetic Landscape

Together, these cumulative data on PAGE4 provided support for the MRK hypothesis. In particular, they indicated that IDP (PAGE4) conformational dynamics can shape cell fates (in this case from androgen-dependent to androgen-independent). We thus turned to Waddington’s epigenetic landscape to gain additional insight into how IDPs may tilt the balance of a system that is robust to external fluctuations yet can switch phenotypes reversibly in response to external perturbations. MYC is a case in point.

**Figure 7:**
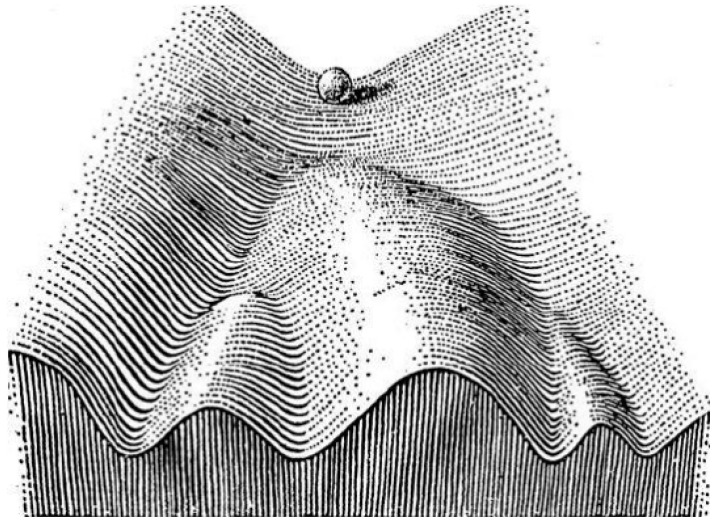
Schematic illustration of Waddington’s epigenetic landscape (adopted and from [50]).

In 1957, Waddington proposed the epigenetic landscape to depict the differentiation of a stem cell [50]. Waddington’s landscape was inspired by Henri Poincare’s dynamical systems theory [51]; therefore, in his metaphor, the concept of “landscape,” from a dynamical systems perspective, represented a high-dimensional space, in which each cell phenotype is considered as an “attractor” and buffered against environmental fluctuations. Conceptually, the differentiation of a stem cell is represented by a ball rolling downhill through a rugged landscape of bifurcating valleys, each representing a possible cell fate. The valleys continue to bifurcate and the ball finally enters one of many sub-valleys at the foot of the hill that form the attractor basin. These sub-valleys represent terminally differentiated states, i.e., cell fates. The cell is held permanently in the terminally differentiated state by high ridges, i.e., valley walls. In Waddington’s terminology, the deeper the valley, the more canalized the cell fate (Figure 7). 

## 9. Insights from Poincare’s Dynamical Systems Theory

In dynamical systems theory pioneered by Poincaré [52], an “attractor” (steady state) represents a set of values of the variables towards which the system evolves from a wide variety of starting conditions and is robust to slight perturbations. As discussed above, proteins in a cell interact to form scale-free PINs and the configuration of such a network is a hallmark of the cell’s phenotype [18]. Thus, in collaboration with Dongya Jia, a smart graduate student with Herbie at Rice, we reasoned that PINs represent dynamical systems that start from context-dependent conditions, develop temporally due to the mutual interactions between the proteins that constitute the PIN, and eventually settle down into “attractors” (stable cell phenotype) [53,54]. Furthermore, we hypothesized that different possible steady states (“attractors”) of a given PIN can be identified by mathematically modeling its dynamics implying that each attractor is associated with a steady-state probability of finding the system in that particular configuration. Thus, it follows that, together, the set of attractors with their relative probabilities of being realized by the system define a high-dimensional “landscape.” Indeed, such a representation has helped realize that single cells can shift from one attractor to another due to noise, without altering the overall population structure. Therefore, such a viewpoint facilitates conceptualizing biological systems from a statistical mechanics perspective where a macrostate (a cell population structure) can correspond to multiple microstates (phenotypic heterogeneity at a single-cell level).

The concept of an “attractor” representing a cell phenotype has also helped elucidate cancer initiation and progression [55,56,57]. In this context, cancer cells are regarded as abnormal cell phenotypes, i.e., “cancer attractors,” and are believed to be stable states of PIN configurations that are latent and not commonly occupied by normal cells. Accesses to such cancer attractors can be enabled by genetic events and/or nongenetic events such as contextual signals and biological noise. For example, loss-of-function mutations in tumor suppressor genes and/or gain-of-function mutations in proto-oncogenes can facilitate the oncogenic properties of cells. Therefore, the probability of gaining access to cancer attractors can be enhanced due to these events and transitions can take place among attractors to benefit cancer cells underscoring the remarkable phenotypic plasticity of these cells [54].

## 10. IDPs and Strange Attractors

**Figure 8:**
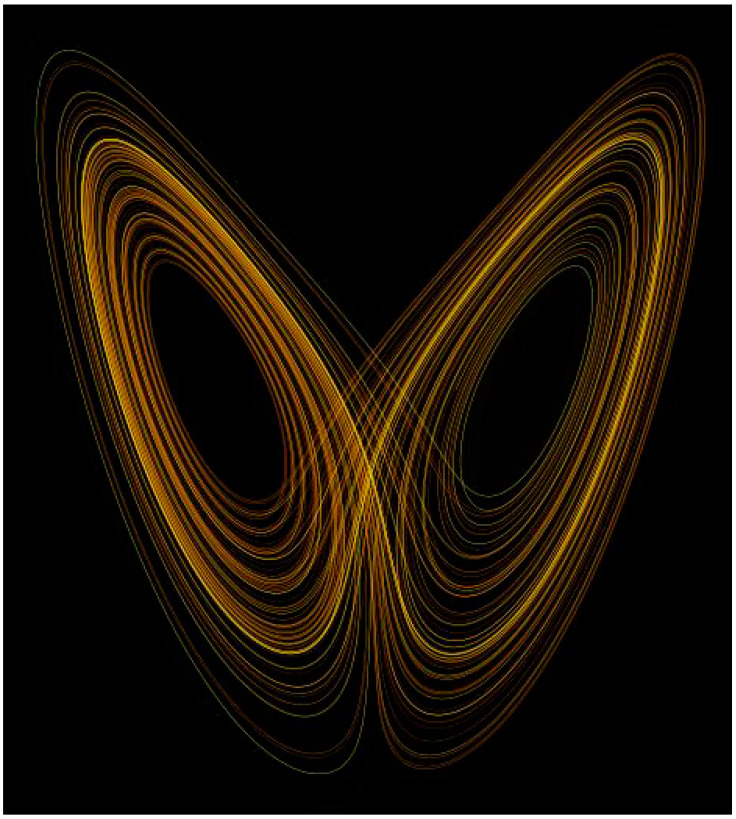
Lorenz attractor potentially describing conformational behavior of an intrinsically disordered protein.

The behavior of certain nonlinear systems whose state evolves with time, and exhibit dynamics that are highly sensitive to initial conditions (the butterfly effect) (Figure 8), can be also described by chaos theory. Because of this sensitivity, which manifests itself as an exponential growth of perturbations in the initial conditions, the behavior of such systems appears random despite being deterministic. Stated differently, their future dynamics are fully defined by their initial conditions, with no random elements involved. This type of behavior is known as deterministic chaos, or simply chaos.

Several years ago, Uversky [58] postulated that IDPs/IDPRs represent “edge of chaos” systems that operate in a region between order and chaos where the complexity is maximal. Thus, even small changes in their environment might generate large and diversified changes defining their exceptional complexity. Therefore, the behavior of an IDP can be described in terms of the strange attractor (e.g., Lorenz attractor), wherein, a system will neither converge to a steady state (do not form a fully ordered state), nor diverge to infinity since they do not behave as completely disordered polypeptide chains either.

## 11. IDPs and the Weak Pinsker Conjecture

Nonetheless, dynamical systems can be completely deterministic. If the system’s position is known at one moment in time, one can predict its position in the future. On the other hand, a dynamical system can be completely random. In other words, even if we know everything about the path up to a certain point, that information will do nothing to help us predict the next step. However, a fundamental feature of dynamical systems, no matter how complex, is that they can be broken up into random and deterministic elements.

In 1960, Pinsker [59] put forth a conjecture (the Pinsker conjecture) that a certain large class of dynamical systems are a mix of a random dynamical system mixed with a deterministic one. This was proven to be incorrect by Donald Ornstein [60,61]. However, in 1977, Thouvenot [62] proposed that Pinsker’s dynamical systems can be the product of a completely random system combined with a system that is *almost* but not completely deterministic. This implies that the simple deterministic system has to have at least a trace of randomness in it. Thus, the weak Pinsker conjecture (as it came to be known after Thouvenot) remained a conjecture for another 30 years.

However, in 2018, Austin [63] provided convincing proof for the weak Pinsker conjecture with support of the “stationary stochastic process,” a mathematical model of a sequence of changing outcomes that are individually random, but with probabilities governed by an underlying law that does not change with time. A key quantity here is the “entropy” of a stationary stochastic process, which quantifies how unpredictable it is. If the entropy is zero, this means that the past of the process determines its future completely and the process is called deterministic. If the entropy is positive, then this quantifies how much “fresh randomness” the process exhibits per unit time on the average over a long period.

From a stationary stochastic process perspective, the Pinsker conjecture seeks to discern whether, through a suitable “encoding,” any stationary (ergodic) process can be separated into two components, running independently of each other, one deterministic and the other independent (purely random). However, the introduction of a trace of randomness, (weaker conjecture) that allows a process with arbitrarily small positive entropy in place of the strictly deterministic component, does hold for all ergodic stationary processes.

From the foregoing, we learned that IDPs can transition from disorder to order (a known protein fold), assume some kind of secondary structure (not necessarily populate a known fold), or remain unfolded (disordered) yet exhibit a preference for a particular conformation within the ensemble of disordered structures (for example, PAGE4). Finally, we also saw examples of some IDPs (for example, neuroligin) that can spontaneously (stochastically) switch between conformational states. Therefore, one can ask, do IDPs that likely represent edge-of-chaos systems abide by the weak Pinsker conjecture? Since these dynamical systems can be completely random or *almost* completely deterministic, can their behavior be modeled to test this possibility? Alternatively, can IDP behavior be modeled using self-organizing criticality [64,65]? This is the second area that I believe is very fertile and likely to break new ground to yield new insights into how IDP behavior influences their function. 

A third area of future research could be the challenge to understand how some IDPs accomplish their functions despite the apparent lack of structure (the so-called fuzzy complex) [66,67,68,69]. In fact, a few years ago, Volodya and I put forth the Janus Challenge to identify (or design) an IDP with catalytic activity. The IDP may also have autocatalytic, de novo synthesis, or self-replicative activity. While an upper limit on the length of the IDP was not specified, we stipulated that it should be at least 30 amino acids long and >90% disordered, as determined experimentally [1]. Perhaps in the next 10 years or so, the Janus Challenge will be met and the IDPs will command increased attention.

## 12. IDPs and Inheritance of Acquired Characteristics – Insights from Lamarckism

A fourth area of fundamental importance, which was pioneered by the late Susan Lindquist’s laboratory at MIT and is at odds with the central dogma, is the intriguing possibility that IDPs can act as vehicles of transgenerational information transfer. While it is now well recognized, largely through the Nobel-Prize-winning work by Prusiner [70], that prions are a paradigm-shifting mechanism of inheritance in which phenotypes are encoded by self-templating protein conformations rather than nucleic acids, work by Lindquist and her colleagues has offered compelling evidence that several IDPs in yeast are functionally equivalent to prions [71]. Thus, the transient overexpression of nearly 50 of these yeast proteins resulted in traits that remained heritable long after their expression returned to normal. These traits were beneficial, had prion-like patterns of inheritance, were common in wild yeasts, and could be transmitted to naive cells with protein alone. Remarkably, however, most inducing proteins were not known prions and did not form amyloid. Instead, they displayed characteristics of nucleic acid-binding proteins with large IDRs and are evolutionarily conserved. These data establish a common type of protein-based inheritance through which IDPs can drive the emergence of new traits and adaptive opportunities. 

More recent work from the Jarosz laboratory (a former associate of Lindquist and now at Stanford) has shown that at least one of these prion-like proteins drives self-assembly into gel-like condensates [72]. However, these proteinaceous particles are not composed of amyloid, yet they are infectious, allowing them to act as a protein-based epigenetic element. Yeast cells harboring such proteins downregulate a coherent network of mRNAs and exhibit improved growth under nutrient limitation. Thus, such nonamyloid self-assembly of RNA-binding proteins in yeast appears to drive a form of epigenetics beyond the chromosome, instilling adaptive gene expression programs that are heritable over long biological timescales. Given the emerging evidence underscoring the capability of IDPs to undergo liquid–liquid phase transitions or coacervation [73] and giving rise to proteinaceous membrane-less organelles [74,75], the ability of intrinsically disordered prion-like particles to self-assemble into gel-like condensates is not surprising. It is very exciting to note that, consistent with the observations in yeast, emerging evidence suggests that the IDP PGL-1, forms aggregate-like structures in germ cells of *C. elegans*. These PGL-1 aggregates are maintained in the germline (inherited) for multiple generations after these animals no longer possess the mutation that originally triggered their formation adding credence to the hypothesis that IDPs can also form self-propagating aggregates in animals and thereby mediate transgenerational inheritance [76].

In light of these fascinating discoveries, it is tempting to speculate that the Cancer/Testis Antigens (CTAs), especially the ones that are located on the X chromosome (CT-X antigens) could play a similar role of protein-based inheritance of acquired characteristics. The CT-X antigens that comprise about half of all known CTAs are remarkably germ cell-specific and appear to have evolved very recently since they are not present in lower animals beyond the primates [77,78]. Furthermore, ~95% of the CT-X antigens are predicted to be IDPs and a majority are predicted to bind DNA [15], a hallmark describing the yeast prion-like IDPs. However, there is no report of their ability to self-assemble yet. Interestingly, among the intrinsically disordered CTAs located on the autosomes (the non-X CT Antigens), some CTAs such as PIWI, Tudor, Maelstrom, and Vasa are involved in the biogenesis of the PIWI-interacting RNAs (piRNAs). These microRNAs can be transgenerationally inherited [79] lending support to the hypothesis that IDPs might be involved in sensing environmental conditions and triggering epigenetic gene expression changes via small RNAs that may lead to increased population fitness of an organism that lives in a dynamic habitat. These findings represent yet another frontier (fifth area) in the IDP field.

## 13. IDPs and Drug Resistance 

Since moving to the City of Hope (COH), I got interested in elucidating how IDPs may modulate drug resistance in cancer. Working with Ravi Salgia’s group and using cisplatin resistance in non-small cell lung cancer as a paradigm, we focused our attention on components of the focal adhesion (FA) complex, namely, paxillin (PXN), integrin β4 (ITGB4), and focal adhesion kinase (FAK). PXN is significantly intrinsically disordered, especially in the N-terminal half of the molecule [45]; thus, it was not surprising that it appeared to occupy a hub position in the PIN that constitutes the FA complex. Critical hubs in the PIN, which follow a power law distribution (are scale-free) [46], contribute to the network’s resilience [80,81]. Thus, we hypothesized that perturbing a critical hub can incapacitate the PIN with downstream physiological consequences as we had proposed in the MRK hypothesis [18]. 

Therefore, to discern the role of the PXN/ITGB4/FAK hub in cisplatin resistance, Atish Mohanty, an imaginative and astute Staff Scientist, and Arin Nam, a talented research associate, teamed up and were able to elegantly demonstrate that the interaction between the three molecules contributes to cisplatin tolerance. Thus, knocking down PXN or ITGB4 increased cisplatin sensitivity in these cells and the double knockdown was more effective than knocking down either alone. Furthermore, a negative feedback loop between ITGB4 and the microRNA mir-1-3p appeared to give rise to a bistable state suggesting that sensitive and tolerant phenotypes can be reversed implying its nongenetic underpinning. Consistently, treating tolerant cells with suberoylanilide hydroxamic acid (SAHA, an HDAC inhibitor) rendered them sensitive and a purified population of ITGB4 low (or high) cells, when cultured separately, recreated the heterogenous population (of cisplatin-sensitive and cisplatin-tolerant cells) [82]. Additional structural biology studies employing multidimensional NMR that are currently underway in collaboration with John should yield further insight on how PXN interacts with ITGB4 and FAK to modulate drug resistance. 

A new dimension that we explored subsequently was the elucidation of the contribution of group behavior (competition and cooperation) to drug resistance in lung cancer [83]. To this end, Arin and Atish used fluorescently marked cisplatin-sensitive and cisplatin-tolerant cells and monitored them in real time. By employing a new phenotypic switching mathematical model with an underpinning of evolutionary game theory that was developed by a group of adventurous and passionate individuals with a mathematical mindset—namely, Supriyo Bhattacharya (COH), Sourabh Kotnala (COH), Srisairam Achuthan (COH), Anusha Nathan (now at Harvard), Herbie, Mohit, his graduate student Kishore Hari, and Rangarajan—we were able to demonstrate that cisplatin-sensitive and cisplatin-tolerant lung cancer cells when cocultured in cisplatin-free and cisplatin-treated environments, exhibit drastically different group strategies in response to environmental changes. While tolerant cells exhibited a persister-like behavior and were attenuated by sensitive cells, sensitive cells “learned” to evade chemotherapy from tolerant cells when cocultured. Further, tolerant cells could switch phenotypes to become sensitive, although high cisplatin concentrations suppressed this switching. Finally, switching cisplatin administration from continuous to intermittent suppressed the emergence of tolerant cells suggesting that intermittent rather than continuous chemotherapy may result in better outcomes in lung cancer. Confirming these results in vivo in a zebrafish model (by Saumya Srivastava and Linlin Guo, in Ravi’s group), further inspired our confidence.

## 14. IDPs and Multicellularity

Multicellularity marks one of the landmark events in the evolution of life. Yet, how this major evolutionary transition [84] occurred >2.5 billion years ago, mechanistically speaking, remains poorly understood. Although multicellularity by definition refers to a state composed of more than one cell, organisms can be described as facultative or obligate multicellular. A major distinction between the two forms is that, in the case of the former, individual cells can become part of a multicellular body in response to environmental conditions, and then can revert to being unicellular (protists) again. They do not rely on being multicellular in order to survive and reproduce. In contrast, when individual cells are obligately part of a multicellular body and cannot survive and reproduce outside of the multicellular body, they represent an obligate organism. Obligate multicellularity is developmentally determined, and not a response to environmental conditions. Therefore, a key step in the evolution of obligate multicellular organisms is the formation of cooperative groups in which the individuals perform specialized functions (differentiate) and become dependent on each other. This requirement underscores the paramount importance of the phenotypic plasticity of the individual cells. 

Although I skirted the issue previously [18,85,86], a quantitative conceptual framework elucidating how IDPs may have played a crucial role in the origin and evolution of multicellularity was lacking. Thus, in collaboration with our colleagues in mathematics led by Sourabh and Supriyo, we built a toy model that embodied a bistable system with two IDPs that transcriptionally autoregulatory their expression but are mutually repressive. This simple model based on stochastic differential equations implements additive noise to account for the role of extrinsic factors influencing the system and demonstrates that, indeed, such a system can sample multiple states (is multipotential). To simulate situations when multipotential cells come together to form a multicellular system, and to discern how such systems would opt a facultative or obligate multicellular form, we considered the role of communication between boundary cells and their extracellular surroundings. We modeled cell–cell and cell–environment exchange by using diffusion-like terms and developed a generalized system of equations for the multicellular model. Briefly, our model shows that (i) a transition from facultative to obligatory can be the result of different boundary conditions, (ii) cell characteristic of being totipotent, pluripotent, multipotent, or fully differentiated may be explained, at least in part, by the boundary effects, and (iii) a pattern may emerge due to the relative position of cells with differing potency indicating spatial organization. This protozoan perspective on the origins of multicellularity sheds new light on how cancer cells that exhibit atavistic behavior adapt to changing environments and evolve fitness strategies. A preliminary account of this model was submitted for presentation at the annual meeting of the American Association for Cancer Research, 2020. Demonstrating how phenotypic plasticity mediated by IDPs is paramount to the origin of obligate multicellularity, will be a fundamental and important discovery (and the sixth area I identify) in the field.

## 15. Conclusions

Like Blobel and Sabatini, we began with a hypothesis and a simple model to highlight the role of IDPs in phenotypic switching. Over the past decade, the model has undergone considerable refinements and grown in scope from cellular transformation to include embryonic development, reprogramming, drug resistance, and the transition of unicellular forms to multicellularity during evolution. Indeed, the serendipitous discovery that PAGE4 is an IDP led us to formulate the MRK hypothesis which provides an elegant conceptual framework supporting the genetic/nongenetic duality of cancer [87], and more generally, for genetic assimilation of acquired characteristics [85]. Nonetheless, the hypothesis has not gained wide acceptance likely due to its nongenetic underpinning. Perhaps, it may even be bantered given how firmly the idea that genotype dictates phenotype is rooted in people’s minds much like the Anfinsen dictum that structure defines function. However, like the famous Cambridge economist John Maynard Keynes said, “*The difficulty lies, not in the new ideas, but in escaping from the old ones.*”

I began my career as a PI at Johns Hopkins when I was >50 years old; obviously, compared to my contemporaries, I suffered a huge setback. I have never held a tenured position or had a major grant to support my ideas, and neither did I receive any accolades or recognitions except a Travel Fellowship from the Fulbright Scholar Program and election as a Fellow of the Royal Society of Biology, UK. However, I did not let this bog me down. On the contrary, I am happy that at least I had the opportunity to pursue a few interesting questions, and if my previous experience in identifying important questions in science (the signal hypothesis and pattern formation during development both of which earned the Nobel Prize) is an indicator of how to discern what is important from that which is ancillary, then some of the areas I have identified in this essay will prove I was right. I also cherish the book, Phenotypic Switching; Implications in Biology and Medicine, that I had the good fortune to coedit with Vidyanand (Vidya) Nanjundiah (CHG, India), Herbie, and Mohit [88]. I also pride in the success the students and postdocs who worked with me have achieved in their own careers and above all, I pride myself for inculcating a culture of questioning and one which defines purpose in science by the quality of the question and its answer, and not by the volume of herd opinion—a culture that Obaid Siddiqi (NCBS, India) fostered.

In summing up, my advice to youngsters contemplating a career in science is that it can be a wild, tumultuous, and unpredictable journey, but one that can be highly adventurous, exciting, and rewarding. *Choose a laboratory you yearn to be in and not because you want to be in a laboratory.* Persevere, be determined, dare to think big, dare to question, and above all, follow your gut. Always remember your teachers and mentors and respect them for all they have done for you. Given the current funding situation (that may be likened to the “tragedy of the commons,” albeit regulated commons), keep in mind that finding a tenure track position is no trivial matter. However, in case you decide to pursue a career in academics and do land a position as a PI, be aware that it comes with enormous responsibilities toward science, society, and your mentees. Always remember to thank your mentees for what they do/did for you. Try to form a bond with them and as much as possible, help them to succeed. 

I pen down by sharing a quote by Albert Szent-Gyorgyi that has been my guiding principle throughout: “*Discovery is seeing what everybody else has seen but thinking what nobody else has thought.*” The IDPs, very likely, were around albeit in their most primitive forms, for at least four billion years [86]. Molecular biologists and crystallographers saw them but dismissed them as being unimportant. Indeed, as Keith Dunker points out in his lectures, several Nobel Prizes have been awarded based on work done on IDPs except that neither the Nobel Committee nor the Laureate was aware of it! Vladimir Uversky saw the IDPs like everyone, but he thought about them like no one did! I inculcated this doctrine and, in my own way, have tried to do so in those who work with me.

## Footnote

*Despite the name and intended definition of IDPs, I do not believe there is a protein that lacks structure. After all, an IDP is not a random coil. Thus, it follows that even an IDP such as PAGE4, which is predicted to be 100% disordered, has conformational preferences that it samples transiently. In fact, a recent study showed a small region of PAGE4 in the N-terminus can even be co-crystalized together with its interacting partner, the poly-(ADP)-ribose polymerase Tankyrase [89]! Thus, IDPs do not breach Anfinsen’s dictum; perhaps, they appear to abide by a “weak” Anfinsen’s dictum analogous to the weak Pinsker conjecture.

## References

[1] Kulkarni P., Uversky V.N. (2018). Intrinsically Disordered Proteins and the Janus Challenge. Biomolecules.

[2] Anfinsen C.B. (1973). Principles that govern the folding of protein chains. Science.

[3] Levinthal C., Debrunner P., Tsibris J.C.M., Munck E. (1969). Mossbauer Spectroscopy in Biological Systems: Proceedings of a Meeting Held at Allerton House.

[4] Prakash K., Patil S.D., Hegde S.N. (1983). Studies on the intestinal disaccharidases of the pigeon. III. Separation, purification and properties of sucrase-isomaltase and maltase-glucoamylase. Arch. Int. Physiol. Biochim..

[5] Prakash K., Patil S.D., Hegde S.N. (1982). Studies on the intestinal disaccharidases of the pigeon II. Subcellular localization and solubilization. Arch. Int. Physiol. Biochim..

[6] Blobel G., Dobberstein B. (1975). Transfer of proteins across membranes. I. Presence of proteolytically processed and unprocessed nascent immunoglobulin light chains on membrane-bound ribosomes of murine myeloma. J. Cell Biol..

[7] Blobel G., Dobberstein B. (1975). Transfer of proteins across membranes. II. Reconstitution of functional rough microsomes from heterologous components. J. Cell Biol..

[8] Blobel G., Sabatini D.D., Manson L.A. (1971). Biomembranes.

[9] Wacker H., Jaussi R., Sonderegger P., Dokow M., Ghersa P., Hauri H.P., Christen P., Semenza G. (1981). Cell-free synthesis of the one-chain precursor of a major intrinsic protein complex of the small-intestinal brush border membrane (pro-sucrase-isomaltase). FEBS Lett..

[10] Prakash K., Fang X.D., Engelberg D., Behal A., Parker C.S. (1992). dOct2, a Drosophila Oct transcription factor that functions in yeast. Proc. Natl. Acad. Sci. USA.

[11] Prakash K., Pirozzi G., Elashoff M., Munger W., Waga I., Dhir R., Kakehi Y., Getzenberg R.H. (2002). Symptomatic and asymptomatic benign prostatic hyperplasia: Molecular differentiation by using microarrays. Proc. Natl. Acad. Sci. USA.

[12] Zeng Y., He Y., Yang F., Mooney S.M., Getzenberg R.H., Orban J., Kulkarni P. (2011). The cancer/testis antigen prostate-associated gene 4 (PAGE4) is a highly intrinsically disordered protein. J. Biol. Chem..

[13] Iakoucheva L.M., Brown C.J., Lawson J.D., Obradović Z., Dunker A.K. (2002). Intrinsic disorder in cell-signaling and cancer-associated proteins. J. Mol. Biol..

[14] Uversky V.N., Oldfield C.J., Dunker A.K. (2008). Intrinsically disordered proteins in human diseases: Introducing the D2 concept. Annu. Rev. Biophys..

[15] Rajagopalan K., Mooney S.M., Parekh N., Getzenberg R.H., Kulkarni P. (2011). A majority of the cancer/testis antigens are intrinsically disordered proteins. J. Cell Biochem..

[16] Takahashi K., Yamanaka S. (2006). Induction of pluripotent stem cells from mouse embryonic and adult fibroblast cultures by defined factors. Cell.

[17] Xue B., Oldfield C.J., Van Y.Y., Dunker A.K., Uversky V.N. (2012). Protein intrinsic disorder and induced pluripotent stem cells. Mol. Biosyst..

[18] Mahmoudabadi G., Rajagopalan K., Getzenberg R.H., Hannenhalli S., Rangarajan G., Kulkarni P. (2013). Intrinsically disordered proteins and conformational noise: Implications in cancer. Cell Cycle.

[19] Dyson H.J., Wright P.E. (2002). Insights into the structure and dynamics of unfolded proteins from nuclear magnetic resonance. Adv. Protein Chem..

[20] Oldfield C.J., Cheng Y., Cortese M.S., Romero P., Uversky V.N., Dunker A.K. (2005). Coupled folding and binding with alpha-helix-forming molecular recognition elements. Biochemistry.

[21] Sugase K., Dyson H.J., Wright P.E. (2007). Mechanism of coupled folding and binding of an intrinsically disordered protein. Nature.

[22] Boehr D.D., Nussinov R., Wright P.E. (2009). The role of dynamic conformational ensembles in biomolecular recognition. Nat. Chem. Biol..

[23] Choi U.B., McCann J.J., Weninger K.R., Bowen M.E. (2011). Beyond the random coil: Stochastic conformational switching in intrinsically disordered proteins. Structure.

[24] Vavouri T., Semple J.I., Garcia-Verdugo R., Lehner B. (2009). Intrinsic protein disorder and interaction promiscuity are widely associated with dosage sensitivity. Cell.

[25] Marcotte E.M., Tsechansky M. (2009). Disorder, promiscuity, and toxic partnerships. Cell.

[26] Ladbury J.E., Arold S.T. (2012). Noise in cellular signaling pathways: Causes and effects. Trends Biochem. Sci..

[27] Eldar A., Elowitz M.B. (2010). Functional roles for noise in genetic circuits. Nature.

[28] Hansen M.M.K., Desai R.V., Simpson M.L., Weinberger L.S. (2018). Cytoplasmic amplification of transcriptional noise generates substantial cell-to-cell variability. Cell Syst..

[29] Pisco A.O., Brock A., Zhou J., Moor A., Mojtahedi M., Jackson D., Huang S. (2013). Non-Darwinian dynamics in therapy-induced cancer drug resistance. Nat. Commun..

[30] Kulkarni V., Kulkarni P. (2019). Intrinsically disordered proteins and phenotypic switching: Implications in cancer. Prog. Mol. Biol. Transl. Sci..

[31] Rajagopalan K., Qiu R., Mooney S.M., Rao S., Shiraishi T., Sacho E., Huang H., Shapiro E., Weninger K.R., Kulkarni P. (2014). The Stress-response protein prostate-associated gene 4, interacts with c-Jun and potentiates its transactivation. Biochim. Biophys. Acta.

[32] Huang C., Wang Y., Li D., Li Y., Luo J., Yuan W., Ou Y., Zhu C., Zhang Y., Wang Z. (2004). Inhibition of transcriptional activities of AP-1 and c-Jun by a new zinc finger protein ZNF394. Biochem. Biophys. Res. Commun..

[33] Lee T., Moran-Gutierrez C.R., Deniz A.A. (2015). Probing protein disorder and complexity at single-molecule resolution. Semin. Cell Dev. Biol..

[34] Gomes G.N., Gradinaru C.C. (2017). Insights into the conformations and dynamics of intrinsically disordered proteins using single-molecule fluorescence. Biochim. Biophys. Acta Proteins Proteom..

[35] LeBlanc S.J., Kulkarni P., Weninger K.R. (2018). Single Molecule FRET: A Powerful Tool to Study Intrinsically Disordered Proteins. Biomolecules.

[36] Iakoucheva L.M., Radivojac P., Brown C.J., O’Connor T.R., Sikes J.G., Obradovic Z., Dunker A.K. (2004). The importance of intrinsic disorder for protein phosphorylation. Nucleic Acids Res..

[37] Basile W., Salvatore M., Bassot C., Elofsson A. (2019). Why do eukaryotic proetins contain more intrinsically disordered regions?. PLoS Comput. Biol..

[38] Mooney S.M., Qiu R., Kim J.J., Sacho E.J., Rajagopalan K., Johng D., Shiraishi T., Kulkarni P., Weninger K.R. (2014). Cancer/testis antigen PAGE4, a regulator of c-Jun transactivation, is phosphorylated by homeodomain-interacting protein kinase 1, a component of the stress-response pathway. Biochemistry.

[39] Kulkarni P., Jolly M.K., Jia D., Mooney S.M., Bhargava A., Kagohara L.T., Chen Y., Hao P., He Y., Veltri R.W. (2017). Phosphorylation-induced conformational dynamics in an intrinsically disordered protein and potential role in phenotypic heterogeneity. Proc. Natl. Acad. Sci. USA.

[40] He Y., Chen Y., Mooney S.M., Rajagopalan K., Bhargava A., Sacho E., Weninger K., Bryan P.N., Kulkarni P., Orban J. (2015). Phosphorylation-induced Conformational Ensemble Switching in an Intrinsically/Testis Antigen. J. Biol. Chem..

[41] Lin X., Roy S., Jolly M.K., Bocci F., Schafer N.P., Tsai M.Y., Chen Y., He Y., Grishaev A., Weninger K. (2018). PAGE4 and Conformational Switching: Insights from Molecular Dynamics Simulations and Implications for Prostate Cancer. J. Mol. Biol..

[42] Lin X., Kulkarni P., Bocci F., Schafer N.P., Roy S., Tsai M.Y., He Y., Chen Y., Rajagopalan K., Mooney S.M. (2019). Structural and Dynamical Order of a Disordered Protein: Molecular Insights into Conformational Switching of PAGE4 at the Systems Level. Biomolecules.

[43] Levy Y., Onuchic J.N., Wolynes P.G. (2007). Fly-casting in protein-DNA binding: Frustration between protein folding and electrostatics facilitates target recognition. J. Am. Chem Soc..

[44] Trizac E., Levy Y., Wolynes P.G. (2010). Capillarity theory for the fly-casting mechanism. Proc. Natl. Acad. Sci. USA.

[45] Neerathilingam M., Bairy S.G., Mysore S. (2016). Deciphering mode of action of functionally important regions in the intrinsically disordered paxillin (Residues 1-313) using its interaction with FAT (Focal Adhesion Targeting Domain of Focal Adhesion Kinase). PLoS ONE.

[46] Barabasi A.L., Albert R. (1999). Emergence of scaling in random networks. Science.

[47] Terada N., Shiraishi T., Zeng Y., Aw-Yong K.M., Mooney S.M., Liu Z., Takahashi S., Luo J., Lupold S.E., Kulkarni P. (2014). Correlation of Sprouty1 and Jagged1 with aggressive prostate cancer cells with different sensitivities to androgen deprivation. J. Cell Biochem..

[48] Shachaf C.M., Kopelman A.M., Arvanitis C., Karlsson A., Beer S., Mandl S., Bachmann M.H., Borowsky A.D., Ruebner B., Cardiff R.D. (2004). MYC inactivation uncovers pluripotent differentiation and tumour dormancy in hepatocellular cancer. Nature.

[49] Rangarajan N., Fox Z., Singh A., Kulkarni P., Rangarajan G. (2015). Disorder, oscillatory dynamics and state switching: The role of c-Myc. J. Theor. Biol..

[50] Waddington C.H. (1957). The Strategy of the Genes.

[51] Slack J.M. (2002). Conrad Hal Waddington: The last Renaissance biologist?. Nat. Rev. Genet..

[52] Poincar’e H. (1890). Sur le problème des trois corps et leséquations de la dynamique. Acta Math..

[53] Mooney S.M., Jolly M.K., Levine H., Kulkarni P. (2016). Phenotypic plasticity in prostate cancer: Role of intrinsically disordered proteins. Asian, J. Androl..

[54] Jia D., Jolly M.K., Kulkarni P., Levine H. (2017). Phenotypic plasticity and cell fate decisions in cancer: Insights from dynamical systems theory. Cancers.

[55] Huang S., Kauffman S. (2013). How to escape the cancer attractor: Rationale and limitations of multi-target drugs. Semin. Cancer Biol..

[56] Huang S., Ernberg I., Kauffman S. (2009). Cancer attractors: A systems view of tumors from a gene network dynamics and developmental perspective. Semin. Cell Dev. Biol..

[57] Li Q., Wennborg A., Aurell E., Dekel E., Zou J.Z., Xu Y., Huang S., Ernberg I. (2016). Dynamics inside the cancer cell attractor reveal cell heterogeneity, limits of stability, and escape. Proc. Natl. Acad. Sci. USA.

[58] Uversky V.N. (2013). Unusual biophysics of intrinsically disordered proteins. Biochim. Biophys. Acta.

[59] Pinsker M.S. (1960). Dynamical systems with completely positive or zero entropy. Soviet Math. Dokl..

[60] Ornstein D. (1970). Bernoulli shifts with the same entropy are isomorphic. Adv. Math..

[61] Ornstein D. (1970). Two Bernoulli shifts with infinite entropy are isomorphic. Adv. Math..

[62] Thouvenot J.-P. (1977). On the stability of the weak Pinsker property. Israel. J. Math..

[63] Austin T. (2018). Measure concentration and the weak Pinsker property. Publ. Math. IHES.

[64] Bak P., Tang C., Wiesenfeld K. (1987). Self-organized criticality: An explanation of the 1/f noise. Phys. Rev. Lett..

[65] Moret M.A. (2011). Self-organized critical model for protein folding. Phys. A Stat. Mech. Appl..

[66] Fuxreiter M., Tompa P. (2012). Fuzzy complexes: A more stochastic view of protein function. Adv. Exp. Med. Biol..

[67] Olsen J.G., Teilum K., Kragelund B.B. (2017). Behavior of intrinsically disordered proteins in protein-protein complexes with an emphasis on fuzziness. Cell Mol. Life Sci..

[68] Arbesú M., Iruela G., Fuentes H., Teixeira J.M.C., Pons M. (2018). Intramolecular Fuzzy Interactions Involving Intrinsically Disordered Domains. Front. Mol. Biosci..

[69] Wang W., Wang D. (2019). Extreme Fuzziness: Direct Interactions between Two IDPs. Biomolecules.

[70] Prusiner S.B. (1998). Prions. Proc. Natl. Acad. Sci. USA.

[71] Chakrabortee S., Meersman F., Kaminski Schierle G.S., Bertoncini C.W., McGee B., Kaminski C.F., Tunnacliffe A. (2010). Catalytic and chaperone-like functions in an intrinsically disordered protein associated with desiccation tolerance. Proc. Natl. Acad. Sci. USA.

[72] Chakravarty A.K., Smejkal T., Itakura A.K., Garcia D.M., Jarosz D.F. (2020). A Non-amyloid prion particle that activates a heritable gene expression program. Mol. Cell.

[73] Uversky V.N., Kuznetsova I.M., Turoverov K.K., Zaslavsky B. (2015). Intrinsically disordered proteins as crucial constituents of cellular aqueous two phase systems and coacervates. FEBS Lett..

[74] Uversky V.N. (2017). Intrinsically disordered proteins in overcrowded milieu: Membrane-less organelles, phase separation, and intrinsic disorder. Curr. Opin. Struct. Biol..

[75] Uversky V.N. (2017). Protein intrinsic disorder-based liquid-liquid phase transitions in biological systems: Complex coacervates and membrane-less organelles. Adv. Colloid. Interface Sci..

[76] Kennedy S.G. https://grantome.com/grant/NIH/R21-AG061850-01A1.

[77] Dobrynin P., Matyunina E., Malov S.V., Kozlov A.P. (2013). The novelty of human cancer/testis antigen encoding genes in evolution. Int. J. Genomics.

[78] Zhang Q., Su B. (2014). Evolutionary origin and human-specific expansion of a cancer/testis antigen gene family. Mol. Biol. Evol..

[79] Sharma U. (2019). Paternal contributions to offspring health: Role of sperm small RNAs in intergenerational transmission of epigenetic information. Front. Cell Dev. Biol..

[80] Barrat A., Barthelemy M., Vespignani A. (2008). Dynamical Processes on Complex Networks.

[81] Rodrigues F.A., da Fontoura Costa L., Barbieri A.L. (2011). Resilience of protein-protein interaction networks as determined by their large-scale topological features. Mol. Biosyst..

[82] Mohanty A., Nam A., Pozhitkov A., Yang L., Srivastava S., Nathan A., Wu X., Mambetsariev I., Nelson M., Subbalakshmi A.R. (2020). A non-genetic mechanism involving the integrin ß4/paxillin axis contributes to chemoresistance in lung cancer. iScience.

[83] Nam A., Mohanty A., Bhattacharya S., Kotnala S., Achuthan S., Hari K., Srivastav S., Guo L., Nathan A., Rangarajan G. (2020). Intermittent therapy can suppress chemoresistance in lung cancer via dynamic phenotypic switching. BioRxiv.

[84] Szathmáry E., Smith J.M. (1995). The major evolutionary transitions. Nature.

[85] Sonnenschein C., Soto A.M., Rangarajan A., Kulkarni P. (2014). Competing views on cancer. J. Biosci..

[86] Kulkarni P., Uversky V.N. (2018). Intrinsically Disordered Proteins: The Dark Horse of the Dark Proteome. Proteomics.

[87] Salgia R., Kulkarni P. (2018). The genetic/non-genetic duality of drug ‘resistance’ in cancer. Trends Cancer.

[88] Levine H., Jolly M.K., Kulkarni P., Nanjundiah V. (2020). Phenotypic Switching: Implications in Biology and Medicine.

[89] Koirala S., Klein J., Zheng Y., Glenn N.O., Eisemann T., Tacer K.F., Miller D.J., Kulak O., Lu M., Finkelstein D.B. (2020). Tissue-Specific Regulation of the Wnt/β-Catenin Pathway by PAGE4 Inhibition of Tankyrase. Cell Rep..

